# An emerging need for developing new models for myocardial infarction as a chronic complex disease: lessons learnt from animal vs. human studies on cardioprotective effects of Erythropoietin in reperfused myocardium

**DOI:** 10.3389/fphys.2014.00044

**Published:** 2014-02-11

**Authors:** Soroush Seifirad

**Affiliations:** ^1^Department of Pediatric Cardiology, Children's Medical Center, Tehran University of Medical SciencesTehran, Iran; ^2^Endocrinology and Metabolism Research Center, Endocrinology and Metabolism Clinical Sciences Institute, Tehran University of Medical SciencesTehran, Iran

**Keywords:** Erythropoietin, myocardial infarction, animal study, clinical study design, modeling and simulation, complex diseases, chronic disease

## Introduction

A large number of studies are currently focused on diagnosis, prevention, and treatment of complex disorders. Animal studies are generally utilized as basic models for *in-vivo* studies and support subsequent clinical trials. Although animal models have so far been fruitful in understanding the mechanism of several diseases and testing the likely prevention and treatment strategies; some of them do not appropriately mimic complex disorders. There is an emerging need for developing new models for chronic complex diseases.

Simplifying a complex system is needed for studying the basis of a complex phenomenon (i.e., Gregor Johann Mendel studies on *Pisum sativum* to discover genetic inheritance roles); however, these models do not reflect all aspects of a complex system (i.e., epistasis or inter/intra-allelic gene suppression). When an animal model is used as the basis of a clinical trial, it is needed to mimic all complex features of the patients.

Myocardial infarction (MI) as a leading cause of mortality and morbidity worldwide has been extensively studied in medicine. MI is an acute phenomenon; however usually it comes after a prolonged period of atherosclerosis which is accompanied by hypoperfusion, hypoxia, and probably degrees of heart failure. As a matter of fact acute MI is the final sequence of the atherosclerosis as a chronic process.

Animal models of MI generally do not completely mimic MI process. Although atherosclerosis models are currently available (Fuster et al., 2012; Getz and Reardon, 2012), usually MI animal models are surgically mimicked by clumping-reperfusion process (Moon et al., 2003, 2006; Prunier et al., 2007; Doue et al., 2008; Roubille et al., 2013b).

Here, as an example, I have tried to discuss the paradoxical positive results of animal studies on protective effects of Erythropoietin (EPO) administration in reperfused myocardium vs. negative consequences in clinical setting.

It seems that the main fault has been occurred in MI modeling for EPO treatment at the level of animal experimental studies, and followed imprecisely without considering the nature of EPO as a cytokine which is naturally secreted in human, and the pathophysiologic state of EPO and EPO receptor in atherosclerotic patients. This article will emphasize on the need of developing new models for MI which mimic its complex character rather than criticizing studies on cardioprotective effects of EPO after percutaneous coronary intervention (PCI) in MI.

## Erythropoietin

Erythropoietin, traditionally an erythropoiesis stimulator cytokine has been shown to have cell protective properties principally by reducing apoptosis (Van Der Meer et al., 2005b; Burger et al., 2006, 2009b). EPO may also enhance neovascularization of ischemic tissue (Van Der Meer et al., 2005a; Hirata et al., 2006). Putative cardioprotection of EPO also has been linked to its anti-inflammatory properties (Burger et al., 2009a). It was also reported as one of the protective factors responsible for hypoxemic preconditioning (Cai et al., 2003). EPO cell protective effects on myocardial ischemia were reported in a series of animals as well as human studies; (Cai et al., 2003; Hirata et al., 2005, 2006; Van Der Meer et al., 2005a; Lipsic et al., 2006a,b), however, the results are quit controversial. Some studies hypothesized that Erythropoietin may protect myocardium by direct action on cardiac myocytes and fibroblasts to modify survival and ventricular remodeling (Parsa et al., 2004). The response of coronary artery endothelium to EPO stimulation by NO production was suggested as another probable protective mechanism of EPO on myocardium (Teng et al., 2011).

## The erythropoietin paradox: a brief review on the main published trials

Preliminary animal studies suggest EPO as an efficient mediator for myocardial protection (Van Der Meer et al., 2004b; Burger et al., 2006, 2009b; Westenbrink et al., 2007a; Lipsic et al., 2008; Prunier et al., 2009). For example, Moon et al., showed that single systemic administration of EPO dramatically decreased infarct size and contractile dysfunction 8 weeks after induction of MI in rats (Moon et al., 2003).

Following animal experiments, randomized clinical trials (RCT) tried to study myocardial protective properties of EPO. Results of HEBE III study in the Netherlands showed an improvement of left ventricular ejection fraction (LVEF) after administration of EPO in a single dose (Belonje et al., 2008). However, in another RCT conducted in France (EPOMI), Single high-dose EPO after reperfusion in patients with ST elevation MI did not decrease infarct size. They noted that EPO treatment was accompanied by temporary effects on left ventricle size and contractility decreasing the incidence of microvascular obstruction (Prunier et al., 2012). No protective effect was reported in preischemic EPO administration in a published study by Kristensen et al. (2005). According to the results of a published study by Voors et al., a single high dose of EPO after a successful PCI for a ST elevation MI did not improve LVEF after 6 weeks. Nevertheless, the EPO administration was correlated to less major adverse cardiovascular events and an admiring clinical safety profile (Voors et al., 2010). Results of REVEAL study by Najjar et al on 222 patients with ST elevation MI showed no reduction in infarct size and surprisingly were linked with increased rates of adverse cardiovascular events. On the other hand, an increase in infarct size among older patients was also anticipated after subgroup analyses of their patients (Najjar et al., 2011). Roubille et al., in a very recent published study concluded that “Early intracoronary administration of a longer-acting erythropoietin analog in patients with acute MI at the time of reperfusion does not significantly reduce infarct size” (Roubille et al., 2013a).

Finally Meta-Analysis of randomized controlled trials revealed that “Erythropoietin in patients with acute MI seems to have no clinical benefit for heart function or reducing infarct size, cardiovascular events, and all-cause mortality. Erythropoietin may not be a choice for patients with acute MI” (Gao et al., 2012). Paradoxical negative results of human studies vs. positive results of animal studies led to the Erythropoietin paradox in reperfused myocardium protection (Roubille et al., 2013b).

## Discussion

The authors of above mentioned published clinical trials have tried to elucidate their negative findings. However, this tended to repetition of studies with more negative results. They have tried to justify their negative observation as consequence of inclusion criteria (i.e., included patients were not limited to proximal LAD occlusion alone), species differences in EPO response, effective dose, reperfusion-EPO infusion therapeutic window (time), and MI sequel evaluation methods (i.e., echocardiography vs. cardiac magnetic resonance imaging, to evaluate infarct size, ejection fraction, and LV function) (Prunier et al., 2007, 2012; Huang et al., 2008; Najjar et al., 2011; Talan et al., 2012; Roubille et al., 2013a,b). A series of above mentioned rationalization have been rejected by later studies (Roubille et al., 2013a).

Atherosclerotic patients usually suffer from degrees of hypoxia, tissue hypoperfussion (i.e., renal hypoperfusion), and anemia which may tend to pathophysiologically high levels of EPO (Gainer, 1987; Shimizu et al., 1988; Van Der Meer et al., 2004a,b, 2008; Westenbrink et al., 2007b). It has been shown that higher levels of EPO are accompanied by higher mortality in a number of patients' subgroups (i.e., Patients with heart failure) (Van Der Meer et al., 2004a). Mortality in these patients mainly originates from cardiovascular accidents. Higher EPO levels reflect poor condition of cardiovascular system and increased likelihood of cardiovascular accidents. Hence, there is a possibility that in case of EPO administration, its role has already been played before MI came to the stage and degrees of EPO resistance is predictable in these patients.

On the other hand, as it has been mentioned above, in the basic animal experiments MI has been produced with no previous hypoxia, no hypoperfusion, no anemia and therefore no high levels of EPO; (Rekhter et al., 1998) hence, It is predictable that externally infused EPO might have protected these animals and had no similarity with the clinical and biochemical state in EPO resistant patients (Bamgbola et al., 2009; Bamgbola, 2012; Chung et al., 2012; Guerrero-Riscos et al., 2012; Mallick et al., 2012; Okonko et al., 2013). Additonally, renal function, renal perfusion, EPO levels and EPO resistance have not been evaluated in above mentioned clinical trials. Additionally there is likelihood of presence of several unknown mechanisms that changes EPO sensitivity and effectiveness in atherosclerotic patients. We should keep in mind that “Only a good beginning makes a good ending”.

## Conclusion

In conclusion the Erythropoietin paradox teaches us a very important lesson: let's have a second look at the animal models of MI. When we are studying extrinsic factors, simple MI models are practical to some instance, but when we are studying a naturally secreted factor such as a cytokine, we should note to the chronic background of acute MI and complex character of our patients compared to the simple animal models constructed for a condition such as MI. The Erythropoietin paradox certified inappropriate modeling of MI as a complex phenomenon.

## Suggestions

New animal models should be developed which mimic chronic complex background of MI. Combination of currently available atherosclerosis models with MI induction techniques could be promising. Recently new state of the art models such as Organ on Chip are introduced; (Huh et al., 2013). Although these new models are at their infancy, they should be considered as future models for *in*-*vivo* studies on chronic complex diseases.
